# Supplementary figures and images for: Integrated taxonomy of black flies (Diptera: Simuliidae) reveals unexpected diversity in the most arid ecosystem of Europe
Source: PLoS One. 2023 Nov 10;18(11):e0293547. doi: 10.1371/journal.pone.0293547 (PMC10637677; doi:10.1371/journal.pone.0293547)

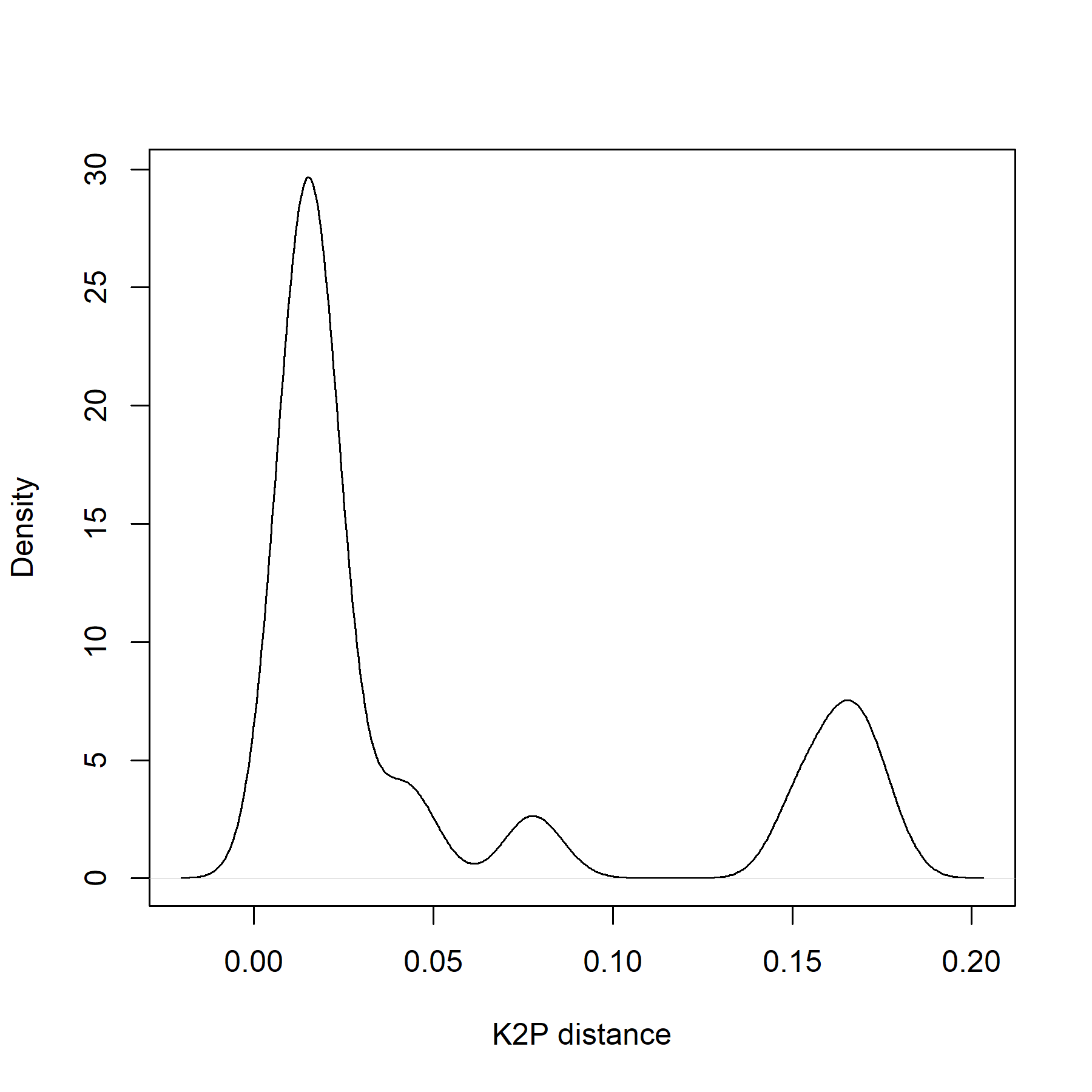

Supplement: S1 Fig — The transition between intra- and interspecific distances is the dip in the density graph, here approximately at 6% or 11%. (TIF) [file pone.0293547.s003.tif]

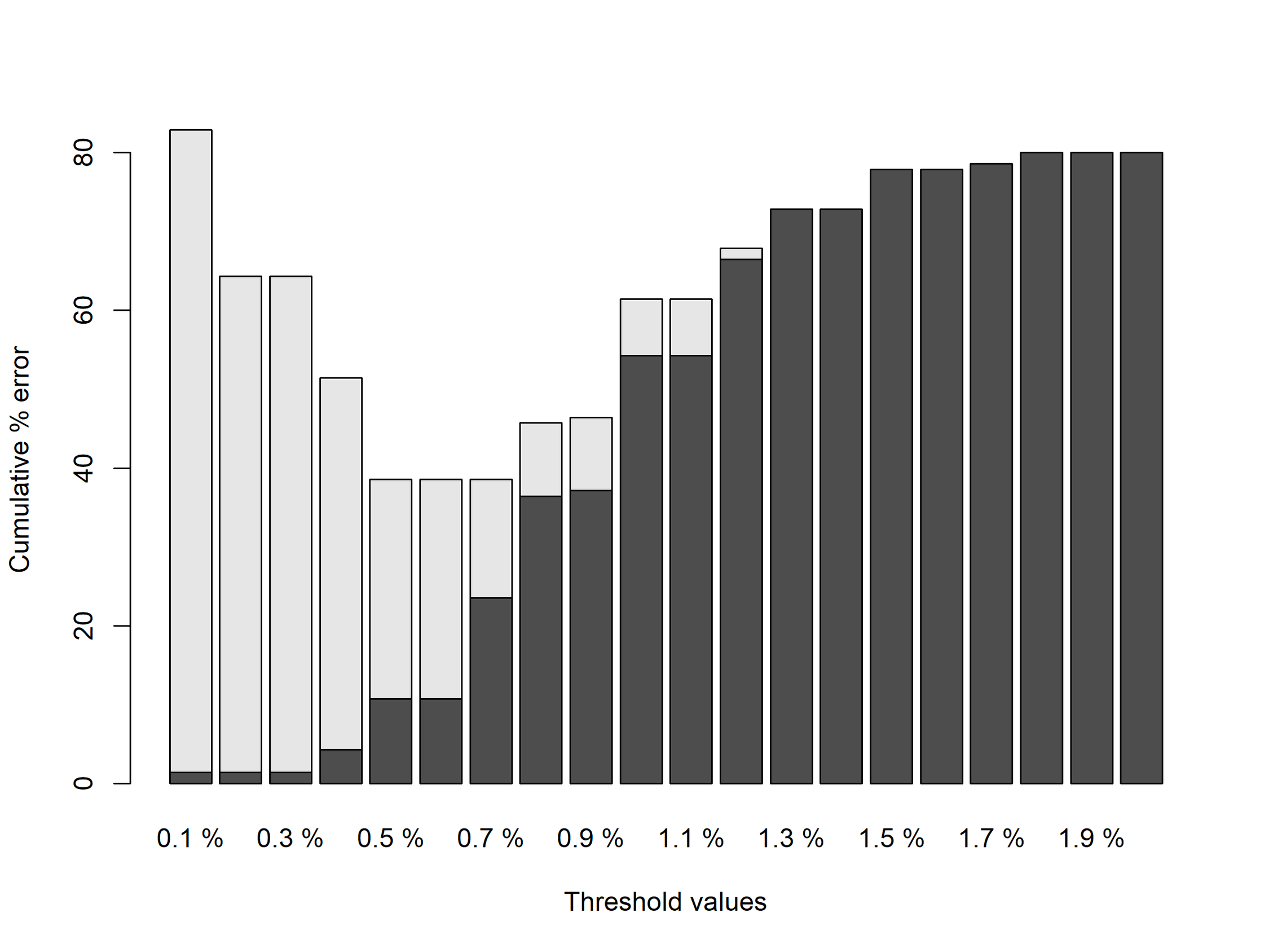

Supplement: S2 Fig — Optimum threshold is between 0.5% and 0.7%. (TIF) [file pone.0293547.s004.tif]

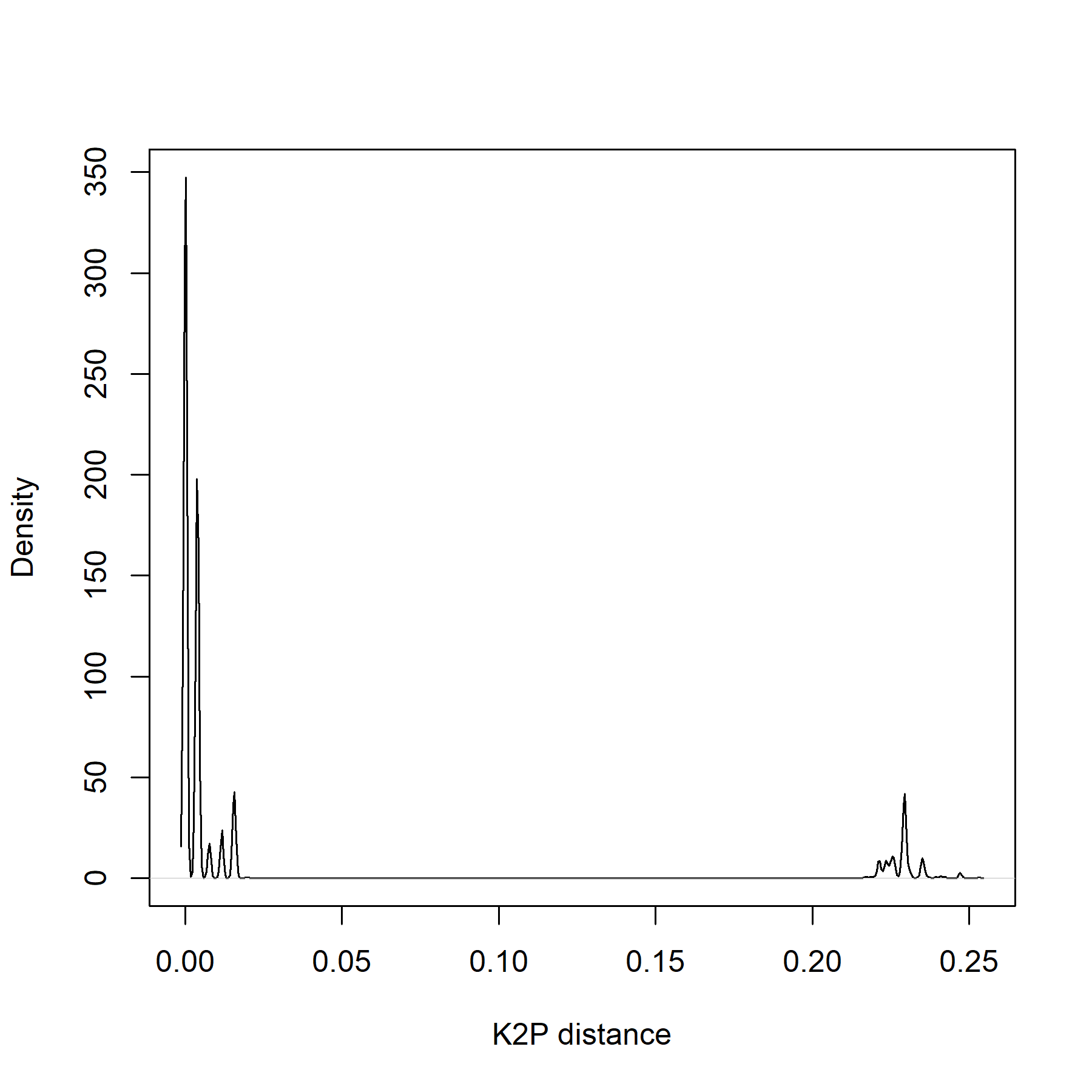

Supplement: S3 Fig — The transition between intra- and interspecific distances is the dip in the density graph. Here several thresholds appear, at low K2P distances, namely 0.12%, 0.52%, 0.92%, 1.27%, and 1.72%. (TIF) [file pone.0293547.s005.tif]

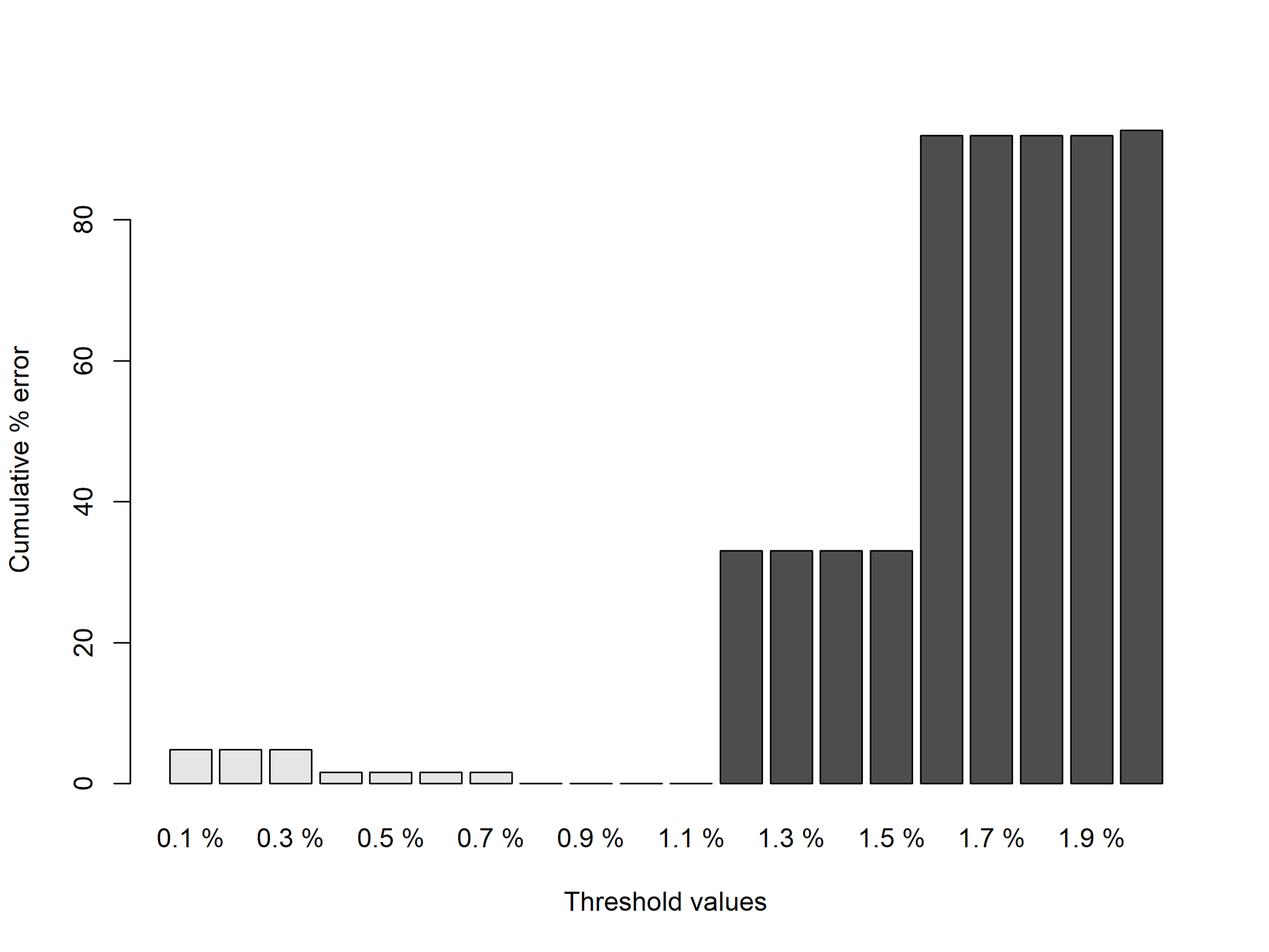

Supplement: S4 Fig — Optimum threshold is between 0.8% and 1.1%. (TIF) [file pone.0293547.s006.tif]
