# Supplementary material for: Integrated taxonomy of black flies (Diptera: Simuliidae) reveals unexpected diversity in the most arid ecosystem of Europe
Source: PLoS One. 2023 Nov 10;18(11):e0293547. doi: 10.1371/journal.pone.0293547 (PMC10637677; doi:10.1371/journal.pone.0293547)
Supplement: S1 File — (DOCX) [file pone.0293547.s007.docx]

**S1 Alternative Language Abstract**

**TITLE AND ABSTRAT IN SPANISH**

**La taxonomía integradora de las moscas negras (Diptera: Simuliidae) revela una diversidad inesperada en el ecosistema más árido de Europa**

La familia Simuliidae incluye más de 2000 especies en todo el mundo. Su uniformidad morfológica dificulta la identificación de las especies, lo que a su vez limita nuestro conocimiento de su ecología y su papel vectorial. Hemos investigado la sistemática de las moscas negras en la zona semi-árida del sureste de la Península Ibérica, un ambiente ecológicamente severo para estos organismos. La captura de adultos de moscas negras en tres tipos de hábitat diferentes (mediante trampas CDC) y en cajas nido de aves, así como la recogida de estadios inmaduros en arroyos de alta salinidad proporcionaron una muestra representativa de las especies presentes. La combinación de estudios morfológicos, cromosómicos, y moleculares (basados en los genes de la subunidad I del citocromo c oxidasa (COI) mitocondrial y del espaciador transcrito interno 2 (ITS2)) reveló la existencia de cinco especies: cuatro comunes (*Simulium intermedium, S. petricolum, S. pseudequinum* y *S. rubzovianum*) y el primer registro europeo de *S. mellah.* Los análisis filogenéticos y de “barcoding gap” revelaron que el marcador ITS2 es clave para identificar las especies, mientras que el marcador COI no proporciona suficiente resolución para diferenciar algunas especies e inferir sus relaciones filogenéticas. También se aportan características morfológicas y cromosómicas para identificar inequívocamente a *S. mellah.* Este trabajo pone de relieve la necesidad de realizar estudios integrados de simúlidos en hábitats ecológicamente extremos para aumentar nuestros conocimientos sobre su distribución, ecología y los riesgos potenciales para la salud pública.
