# Supplementary material for: Integrated taxonomy of black flies (Diptera: Simuliidae) reveals unexpected diversity in the most arid ecosystem of Europe
Source: PLoS One. 2023 Nov 10;18(11):e0293547. doi: 10.1371/journal.pone.0293547 (PMC10637677; doi:10.1371/journal.pone.0293547)
Supplement: Attachment — Submitted filename: Response to Reviewers.docx [file pone.0293547.s008.docx]

September 17th, 2023

Dr. James Lee Crainey

Academic Editor in PLOS ONE

Reference: Integrated taxonomy of black flies (Diptera: Simuliidae) reveals unexpected diversity in the most arid ecosystem of Europe" (Manuscript ID: PONE-D-23-21797)”.

Dear Dr. James Lee

Thank you for your e mail dated August 7th, 2023 regarding our manuscript PONE-D-23-21797. We are delighted that the manuscript has been deemed acceptable for publication following suitable revision. I am including the revised version of the manuscript with this letter (uploaded as a "Revised Manuscript with Track Changes”). We have indicated the modifications made with Track Changes. We have summarized the comments of the reviewers and our response to them in this letter. In addition, the length of the manuscript has been reduced by 2 pages as requested by the Editor. The journal requirements requested have been also met, such as expanding the acronym “MCIN/AEI” (as indicated in the financial disclosure) so that it states the name of our funders in full: *Ministerio de Ciencia e Innovación (MCIN)/Agencia Estatal de Invetigación (AEI).* We wish to make a change in “Data Availability statement” as our data (accession number for each sequence) will be available in GenBank after acceptance. We have included two new references in the manuscript which have been added to the list of references. Finally, all figures have been loaded with better quality and figure 8 has been redone after calculating the maximum intraspecific and minimum interspecific distances.

The authors certify that this material has not and will not be offered elsewhere for possible publication, as long as it is under consideration by PLOS ONE. All authors of this manuscript agreed to allow me to submit revisions on their behalf in the original cover letter. In essence, we have followed all reviewer’s and Editor suggestions and amended our paper to this effect. We hope that this new version will be acceptable for publication in PLOS ONE.

Thank you in advance for taking the time to consider this manuscript.

Sincerely yours,

Ignacio Ruiz-Arrondo

**Journal Requirements**

2. We suggest you thoroughly copyedit your manuscript for language usage, spelling, and grammar. If you do not know anyone who can help you do this, you may wish to consider employing a professional scientific editing service.

Authors: *Language usage, spelling, and grammar of the manuscript has been carefully reviewed by Dr. Peter Adler, who is a native English speaker and co-author of this manuscript.*

3. In your Methods section, please provide additional information regarding the permits you obtained for the work. Please ensure you have included the full name of the authority that approved the field site access and, if no permits were required, a brief statement explaining why.

Authors: *Done*

4. Please expand the acronym “MCIN/AEI” (as indicated in your financial disclosure) so that it states the name of your funders in full. This information should be included in your cover letter; we will change the online submission form on your behalf.

Authors: *the full name of MCIN/AEI is Ministerio de Ciencia e Innovación (MCIN)/Agencia Estatal de Invetigación (AEI). This is also written in the new version of the manuscript.*

5. We note that you have stated that you will provide repository information for your data at acceptance. Should your manuscript be accepted for publication, we will hold it until you provide the relevant accession numbers or DOIs necessary to access your data. If you wish to make changes to your Data Availability statement, please describe these changes in your cover letter and we will update your Data Availability statement to reflect the information you provide.

Authors: *We wish to change the “Data Availability statement” as our data (accession number for each sequence) will be available in GenBank after acceptance of the manuscript.*

6. We are unable to open your Supporting Information file Supporting information.7z. Please kindly revise as necessary and re-upload.

Authors: *We have re-uploaded the Supporting Information in a zip folder.*

7. Please review your reference list to ensure that it is complete and correct. If you have cited papers that have been retracted, please include the rationale for doing so in the manuscript text or remove these references and replace them with relevant current references. Any changes to the reference list should be mentioned in the rebuttal letter that accompanies your revised manuscript. If you need to cite a retracted article, indicate the article’s retracted status in the References list and also include a citation and full reference for the retraction notice.

Authors: *Reference list reviewed. In addition, we have included two new references in the manuscript which have been added to the list of references:*

*-Nei, M., & Tajima, F. (1981). DNA polymorphism detectable by restriction endonucleases. Genetics, 97(1), 145-163.*

*-Nei, M. (1987) Molecular evolutionary genetics. New York: Columbia University Press.*

**Review Comments to the Author**

**Reviewer #1**

This study using morphology, cytology and molecularly (COI and ITS2 sequences) to examine diversity of black flies in Tabernas Desert, Spain. The information provided in the present study is very interesting. I have some comments for author to consider.

Authors: *Thank you for your positive feedback.*

- Line 376, barcoding gap, because the barcode gap analyses based on localMinima and threshOpt methods revealed two very different values (>10x differentiations). It might be useful to use additional method such as those available in TaxonDNA software (Meier et al., 2006) which author used for Best Close Match (BCM) species identification in the present study. Because the successful identification based on the BCM method rely on the threshold value, thus, using inappropriate threshold value can hugely affect the species identification. Also, it will be useful to use additional method for species identification such as Best Match method in TaxonDNA.

Authors: *Thanks for your valuable comment, we agree with the importance of the threshold value and we add an additional method of calculation based on TaxonDNA (please, see lines 279-282 in the version with Tracked changes). We found better identification results for COI but not for ITS2*, *which have been added to the new version of the manuscript (please, see lines 402-404, 469-471 and Table 3 in the version with Tracked changes). Regarding the use of Best Match, we chose not to include it in the manuscript because it assigns the species name from its barcode that best matches, regardless of how similar the query and barcode sequences are. Moreover, the results were quite similar to those obtained by Best Close Match, so we finally decided not to include it in order not to overload this section. However, if the reviewer or editor finally considers including it, we have no objection to include it.*

- Line 386 – 387, It will be very useful to provide range of intraspecific and interspecific genetic distances for all species included in this study.

Authors*: We agree with the reviewer. Accordingly, we provide the suggested information (please, see lines 393-394, 414-415 and Table 3 in the version with Tracked changes). We also noticed that the result of intra- and interspecific distances was not accurately described, as we want to refer to maximum intraspecific and minimum interspecific distances, which are key for barcoding gap analysis. We made some changes in the manuscript to correct it (please, see lines 404-405, 420 and Figure 8 and its caption in the version with Tracked changes).*

- Table 3, In my opinion, the table is easier to read if the same species is report within a single row for each gene. Therefore, I suggest that under the “Identification” column, it can be divided into four subheadings, i.e. correct (%), incorrect (%), ambiguous (%) and no id (%).

Authors: *We modified Table 3 as requested in order to see each species in a single row for ease of reading. We also added intra and interspecific distances to the table, as suggested in the previous comment (please, see Table 3).*

- Line 391, I am surprise that the number of haplotype is very low for the ITS2 sequences but the haplotype diversity value is fairly high. According to line 394, total specimens for the ITS2 sequences are 124 individuals but only 19 haplotypes were identified. However, the haplotype diversity reported is 0.713. Please check this again. Also, it will be useful to report range of intraspecific and interspecific genetic distance for the ITS2 sequences.

Authors: *We double-checked the result, and it is correct. Haplotype diversity, calculated using Nei and Tajima's (1981) method, is defined as one minus the sum of the squared relative frequencies of each haplotype, multiplied by the sample size divided by the sample size minus 1.*

*In this formula, ‘pi’ is the (relative) haplotype frequency of each haplotype in the sample and n is the sample size (*[*Fan*](https://doi.org/10.1111/2041-210X.13643) *et al. 2021).*

*Therefore, for the ITS2, we have 19 haplotypes with the following frequencies: 1, 1, 61, 1, 7, 2, 2, 1, 1, 24, 10, 5, 1, 1, 1, 1, 2, 1, 1. When we calculate one minus the sum of their squared relative frequencies (0,292), we obtain 0,7077, which multiplied by 124 divided by 123, we get 0,713.*

*We did not specify in the previous version the method followed for such calculations, which it is now detailed (please see lines 287-288 in the version with Tracked changes).*

*The results regarding haplotype diversity are intriguing and deserve deep exploration, but additional analysis and considerations are necessary to make accurate predictions.*

*References:*

*-*Nei, M., & Tajima, F. (1981). DNA polymorphism detectable by restriction endonucleases. *Genetics*, *97*(1), 145-163.

*-*Fan, P., Fjeldså, J., Liu, X., Dong, Y., Chang, Y., Qu, Y., ... & Lei, F. (2021). An approach for estimating haplotype diversity from sequences with unequal lengths. *Methods in Ecology and Evolution*, *12*(9), 1658-1667.

**Reviewer #2**

The authors used morphological, chromosomal, and molecular methods to investigate the systematics of black flies in a semi-arid area of the Iberian Peninsula. The rationale is that the Iberian Peninsula is atypical of where black flies breed, therefore, has not been adequately studied. Adult black flies were collected in different ecological habitats using CDC traps, sticky traps in man-made bird nests and pre-imago stages at breeding sites. The morphological and cytological methods employed standard methods and the molecular analysis targeted the mitochondrial cytochrome C oxidase subunit I and the internal transcribed spacer 2 (ITS2) that involved barcoding gap and phylogenetic analyses. Five species of black flies Simulium intermedium, *S. petricolum*, *S. pseudequinum*, *S. rubzovianum* and *S. mellah* were identified, the latter being the first to be recorded in Europe. The ITS2 was found to be the best for species identification and the establishment of the black flies' phylogenetic relationships.

The manuscript is technically sound and the data support the conclusions. The statistical analysis is OK except for the cytotaxonomic analysis where I was expecting the use of Hardy-Weinberg equation to estimate allele frequencies and therefore, the veracity of the S. mellah as a species population. The authors have fully complied with the data availability.

Authors: *Thank you very much for your positive feedback.*

*We respectfully note that Hardy-Weinberg calculations cannot be applied to our data because the inversions (alleles) are fixed (i.e., 100%), except one polymorphic inversion (IIL-52), which was represented in only one homologue in one individual larva. Thus, the polymorphism data on which Hardy-Weinberg calculations would be performed are not possible, either because the inversions are not polymorphic or the single polymorphism represented in only one larva does not meet statistical requirements for calculations. We note that the chromosomal banding pattern, which is unique among all known species in the subgenus, is entirely consistent with* *all other known populations of* S. mellah*, including material from near the type locality; conclusions of conspecificity are, therefore, justified.*

Comments.

I. Keywords. I suggest that 'Iberian Peninsula' 'black flies' and 'diversity' are inserted, and 'aridity' be removed. Both COI and ITS2 be written in full.

Authors: *Thank you for the suggestion. We understand that "black flies" and "diversity" do not have to be included as keywords as they appear in the title of the manuscript and including them would be losing the opportunity to give importance to other key words. As suggested by the reviewer, "Iberian Peninsula" has been included, the COI and ITS2 acronyms have been developed and "aridity" has been removed.*

2. In demonstrating diversity, it would have helped if the spatial distribution of the species in the area was shown.

Authors: *The reviewer's suggestion is interesting, and we are willing to do it if the Editor thinks it is appropriate. Although the authors do not understand how the spatial distribution of species in our case (a study area of moderate size) can demonstrate diversity. Furthermore, we believe that the manuscript is already long and that doing what the reviewer requests would probably require several images (or one that is too complex). We understand that the other images included both in the main text and in the supplementary text are more important than this one.*
